# Source-to-Output Integration of Archived Evidence from an Immunoregulatory-Conditioned Human PBMC Extracellular Preparation

**DOI:** 10.3390/cimb48100972

**Published:** 2026-09-22

**Authors:** Ramón Gutiérrez-Sandoval, Francisco Gutiérrez-Castro, Natalia Muñoz-Godoy, Ider Rivadeneira, Andy Lagos, Jordan Iturra, Francisco Krakowiak, Ignacio Muñoz, Cristián Peña-Vargas, Andrés Toledo

**Affiliations:** 1 OGRD Alliance LLC, Orlando, FL 32827, USA; sourcing@ogrdalliance.org (C.P.-V.); ops@ogrdalliance.org (A.T.); 2 Department of Cancer Research, Flowinmunocell-Bioexocell Group, 08028 Barcelona, Spain; servicios@flowinmunocell.cl (F.G.-C.); contacto@flowinmunocell.cl (N.M.-G.); 3 Department of Outreach and Engagement Programs, OGRD Consortium, Charlestown KN0802, Saint Kitts and Nevis; iderlautaro@gmail.com (I.R.); lagosandy@gmail.com (A.L.); jiconsultant@ogrdconsorcio.com (J.I.); kinesiologo@recell.cl (I.M.); 4 Histopathology Laboratory, Bioclas, Concepción 4030000, Chile; fkconsultant@ogrdconsorcio.com

**Keywords:** retrospective analytical integration, human PBMC-derived culture, immunoregulatory conditioning, multicomponent extracellular preparation, evidence provenance, extracellular-vesicle-associated components, source-to-output framework, evidence boundary

## Abstract

Background: Sequential cytokine conditioning of human peripheral blood mononuclear cell (PBMC)-derived cultures can generate complex extracellular outputs, but retrospective development archives frequently preserve heterogeneous analytical records at different levels of resolution. Methods: This retrospective descriptive study integrated the complete set of interpretable summary-level records available within one documented IL-4/GM-CSF-supported differentiation and subsequent IL-10/TGF-β1 conditioning workflow. This study was designed to evaluate provenance, analytical coherence, and evidence boundaries; unavailable raw, replicate-level, or implementation-level information was not reconstructed. Results: A single represented archived Annexin V/7-AAD output comprised 26,071 events with 99.66% viability. The recovered preparation was represented by an archived heterogeneous particle-associated signal with a predominant region at approximately 170–240 nm and an archived immunoblot record reporting CD9, CD63, CD81, and TSG101 detection. An independent aggregate record labeled as representing ten conditioned cultures reported mean final-environment values of 25,320.16 pg/mL IL-10, 659.35 pg/mL IL-4, 99.61 pg/mL IL-6, and 3.01 pg/mL TNF-α. Because IL-10 and IL-4 were conditioning inputs, these values were not interpreted as de novo cytokine secretion. Conclusions: This study establishes a provenance-controlled method for integrating heterogeneous archived evidence without conflating experimental units, assigning unsupported vesicle biogenesis, or converting compositional observations into functional claims. The resulting analytical baseline defines dedicated next-stage questions for fraction-resolved composition and prospective functional validation.

## 1. Introduction

Dendritic cells and related antigen-presenting-cell populations integrate antigenic, co-stimulatory, inhibitory, cytokine-derived, and metabolic inputs to determine whether immune encounters favor activation, restraint, or peripheral tolerance [1]. Human PBMC- and monocyte-derived culture systems are therefore useful experimental platforms for examining how sequential environmental cues influence source-cell state and extracellular output [2,3,4,5]. IL-4 and granulocyte-macrophage colony-stimulating factor (GM-CSF) are widely used to support monocyte-to-dendritic-cell differentiation, whereas IL-10 and transforming growth factor beta 1 (TGF-β1) can favor regulatory-associated states [2,6,7]. Importantly, a regulatory-associated source state does not by itself demonstrate stable or antigen-specific tolerogenic function, which requires dedicated phenotypic and functional testing [6,7].

Extracellular material recovered from conditioned cultures may contain vesicles, non-vesicular extracellular particles, membrane-derived fragments, lipoprotein-associated structures, soluble proteins, cytokines, and other lipid–protein assemblies [8,9,10]. Current extracellular-vesicle guidance therefore favors method-explicit and biogenesis-neutral terminology when product purity and endosomal origin have not been established [9]. Orthogonal evidence is particularly important because particle size and canonical EV-associated proteins can support the presence of a vesicle-associated component without establishing that the complete recovered output is a purified exosome product [8,9,10].

Human tolerogenic dendritic-cell studies have established several relevant biological layers. IL-10-dependent molecular programs have been characterized in human monocyte-derived tolerogenic dendritic cells [2]; other human protocols have shown stable regulatory phenotypes and altered T-cell responses [7,11]. At the extracellular-output level, Elsayed et al. demonstrated that exosomes purified from regulatory human monocyte-derived dendritic cells generated with TGF-β/IL-10 can modulate recipient dendritic cells and T cells in vitro [12]. These studies provide strong human precedent for source-state-dependent immune regulation, but they address prospectively defined cellular or purified-vesicle systems with experimental designs that differ from retrospective integration of a complete heterogeneous extracellular output.

These human studies also illustrate an important distinction between source-cell phenotype, extracellular-product identity, and downstream immune function. A regulatory-associated dendritic-cell state can be defined by transcriptional, phenotypic, and responder-cell criteria, whereas an extracellular preparation requires its own compositional and provenance framework before activity can be attributed to it. Purified vesicle studies can then address recipient-cell effects with a level of product definition that is not automatically transferable to heterogeneous, complete extracellular outputs. Keeping these evidentiary layers separate is particularly important when the available material originates from a development archive rather than from a prospectively harmonized experimental series [2,7,9,11,12].

The same distinction applies to extracellular-vesicle nomenclature. Detection of particle-associated material together with proteins commonly associated with EVs can support the presence of an EV-associated component, but it does not by itself resolve the relative contribution of vesicular, non-vesicular, soluble, or lipoprotein-associated fractions. Contemporary guidance therefore places value on orthogonal characterization, explicit reporting of the analytical unit, and restraint in assigning biogenesis when the required ultrastructural, fraction-resolved, or raw-instrument evidence is unavailable [8,9,10,13,14,15]. For a retrospective integration, methodological rigor consequently depends as much on preserving what the archive cannot establish as on summarizing what it does preserve.

A methodological gap therefore remains between studies that characterize conditioned immune cells, studies that isolate defined extracellular-vesicle fractions, and studies that directly test immune function. Legacy and retrospective development archives may contain analytical records generated at different stages, with different experimental units and unequal levels of raw-data preservation. There is no widely adopted framework for integrating such records while simultaneously preserving source provenance, preventing pseudoreplication across analytical domains, avoiding unsupported vesicle-biogenesis assignments, and preventing summary-level compositional observations from being converted into functional claims [16,17,18,19,20].

This gap is not limited to extracellular-vesicle research. Translational development programs frequently generate evidence sequentially: source-cell quality may be documented at one stage, extracellular recovery at another, and biochemical or cytokine-associated summaries at later stages. When those records are revisited retrospectively, the temptation is to treat all measurements as if they originated from one synchronized experiment. Doing so can create pseudoreplication, obscure the difference between process inputs and biological outputs, and inflate the apparent resolution of the underlying evidence [17,18,19,20,21,22]. A defensible retrospective framework must therefore preserve both provenance and analytical asymmetry across domains.

The present study addresses this specific gap. Its predefined question is not whether the recovered preparation is functionally tolerogenic or whether a specific extracellular-vesicle biogenesis pathway can be demonstrated. Rather, the question is whether the analytical information preserved across a sequentially conditioned human PBMC source-to-output archive can be integrated into a coherent, auditable analytical object without exceeding the evidentiary resolution of the underlying records. This study therefore focuses on provenance, analytical-unit preservation, a biogenesis-neutral operational identity, and explicit separation between observations supported by the archive and prospective biological questions. The resulting source-to-output evidence architecture is summarized schematically in Figure 1.

## 2. Materials and Methods

### 2.1. Study Design, Archive Scope, and Inclusion Rule

This retrospective, descriptive study integrated analytical outputs from one documented human PBMC-derived source-programming workflow. The available archive was reviewed by analytical domain, and every record that could be unambiguously linked to the documented source-to-output workflow and retained an interpretable analytical unit was included. No record was excluded on the basis of the direction, magnitude, or apparent favorability of its result. Each analytical domain retained its source-specific experimental unit; no cross-domain pooling was performed.

Archive completeness was defined as completeness of the records preserved for the present retrospective integration, not as completeness of the original experimental workflow. Information not retained in the archive, including selected implementation parameters, original instrument files, and replicate-level cytokine values, was classified as unavailable rather than estimated, reconstructed, imputed, or replaced by surrogate values [17,18,21].

For integration purposes, each record was classified according to three attributes: (i) its biological position within the source-to-output sequence, (ii) the analytical unit explicitly preserved in the archive, and (iii) the maximum interpretation supportable at that resolution. Cross-domain consistency was evaluated relationally rather than statistically; evidence from one domain was not used to manufacture replicate structure for another. This rule was applied uniformly to source-culture viability, particle-associated information, the archived immunoblot record, and the final-environment cytokine summary. The resulting framework therefore functions as an evidence-provenance map rather than as a reconstructed prospective dataset. The archive-selection and analytical-provenance matrix is provided in Supplementary Table S1.

### 2.2. Cellular Material and Ethical Provenance

Established, de-identified, research-grade human PBMC-derived precursor cultures were maintained within the archived research program under internal study culture code FIC-BX-hPBMC-IRC-2026-01 for research use only and under controlled in vitro laboratory conditions. The cultures and associated analytical records were pre-existing and were used exclusively for retrospective analytical integration and extracellular-preparation characterization. The archive did not preserve donor-level identifiers or a reliable donor count; accordingly, the number of unique donors and the relationship between donor number and culture number cannot be reconstructed and are not inferred in this study. The designation “ten conditioned cultures” used for the cytokine record refers to the archived analytical unit and must not be interpreted as ten donors.

The extracellular preparation evaluated in this study corresponds to the recovered output of the documented source-programming and extracellular-recovery workflow. No new human biological samples were collected for this study, no intervention involving human participants was performed, no identifiable donor information or associated clinical data were accessed, and no novel human cell line was generated or genetically modified by the authors. Under the institutional research-governance framework, this study was determined not to constitute human-subject research requiring ethics review, consistent with the U.S. Federal Policy for the Protection of Human Subjects (45 CFR Part 46) and the definition of “human subject” in 45 CFR §46.102(e)(1) [23].

### 2.3. Source Programming and Cytokine Conditioning

The archive documented a sequential research procedure comprising IL-4/GM-CSF-supported differentiation followed by IL-10/TGF-β1 regulatory conditioning, recovery of conditioned medium, serial clarification, and size-based extracellular-fraction recovery [24,25]. The preserved records establish the cytokine classes, order of exposure, and biological stages required for source-to-output interpretation. Exact cytokine concentrations, complete exposure durations, medium composition, culture density, exchange schedule, and precise timing of extracellular-material collection were not retained in the retrospective archive and therefore were not reconstructed. IL-10 and IL-4 are treated throughout this manuscript as process inputs when interpreting the final extracellular-environment record. The stage-level workflow and corresponding archival resolution are summarized in Table 1. Additional detail on the preserved workflow and method-class sequence is provided in Supplementary Table S2 and Supplementary Figure S1.

### 2.4. Archived Source-Culture Flow Record

Viability was represented by one archived Annexin V-PE/7-aminoactinomycin D output acquired on an FACSAria III Cell Sorter [26]. The preserved summary comprised 26,071 events distributed across viable, early-apoptotic, late-apoptotic, and necrotic quadrants. Original FCS files, compensation matrices, and complete gating templates were not retained. Separate archived summaries contained two parent-gate regions and CD34/CD38-defined secondary regions. Because the original gating hierarchy and phenotypic validation records were unavailable, these regions are retained only as Population 1 and Population 2 in the Supplementary Materials and are not interpreted as defined cellular phenotypes in the main analysis.

### 2.5. Extracellular Recovery and Archive Resolution

Conditioned medium was recovered after the sequential source-programming stage. The archive documented serial clarification followed by a size-based extracellular-fraction recovery step [27]. Exact centrifugation forces and durations, filtration specifications, molecular-weight or size cutoffs, concentration factors, recovery volumes, and storage parameters were not preserved at a level sufficient for reproducible reconstruction and are therefore not reported as inferred values. The resulting complete recovered preparation was carried forward as the common analytical object for the archived particle and protein records and for integration with the final-environment cytokine record.

### 2.6. Archived Particle Record

Particle-associated characteristics were represented by an archived NanoSight/NTA summary [28]. The preserved record described a heterogeneous signal with a predominant region at approximately 170–240 nm. Original NanoSight acquisition files, acquisition settings, particle-concentration outputs, and replicate-level measurements were not retained and are not available for retrospective reanalysis. The particle record is therefore used qualitatively to support the presence of particle-associated material; it does not establish particle concentration, quantitative size dispersion, or vesicle biogenesis.

### 2.7. Archived Immunoblot Record

The archived immunoblot record reported evaluation and detection of CD9, CD63, CD81, and TSG101 in the recovered extracellular preparation [29]. Uncropped immunoblot images, antibody metadata, loading controls, densitometry, and replicate-level records were not preserved. The record is therefore interpreted as qualitative evidence for an EV-associated protein component and is not used to establish protein abundance, product purity, or an exosome-specific identity.

### 2.8. Archived Final-Environment Cytokine Record

An independent archived multiplex bead-immunoassay summary, processed with FCAP Array, was labeled as representing ten conditioned cultures [30]. The preserved aggregate values included IL-10, IL-4, IL-6, and TNF-α. Culture-level measurements and variability estimates were not retained. Consequently, the reported values cannot support retrospective calculation of standard deviations or inferential testing without generating unsupported replicate-level data. IL-10 and IL-4 were conditioning inputs and are interpreted strictly as final conditioned extracellular-environment descriptors; they are not interpreted as evidence of de novo secretion by the source cultures.

### 2.9. Analytical Integration and Statistical Treatment

The integration reports the archived arithmetic means from the cytokine summary and the directly documented event percentages from the represented viability record. Because experimental units differ across domains and replicate-level values were not available across the archive, no cross-domain inferential statistics, variance estimates, confidence intervals, or reconstructed standard deviations were generated [20,21,22]. The analytical objective is provenance-controlled integration at the resolution preserved in each source record.

### 2.10. Use of Generative AI and AI-Assisted Technologies

AI-assisted tools were used for editorial support during manuscript preparation. They were not used to generate experimental data or results. All content was reviewed and approved by the authors, who take full responsibility for the final manuscript.

## 3. Results

### 3.1. Archive Composition and Experimental-Unit Separation

The integrated archive comprised four analytical domains: one represented source-culture viability output; an archived particle-associated summary of the recovered preparation; an archived band-level protein summary of the recovered preparation; and an independent aggregate final-environment cytokine summary labeled as ten conditioned cultures. These records were not treated as biological replicates of one another. Their integration is relational: each record is linked to the same documented source-to-output workflow while retaining its original analytical unit. The provenance, analytical units, preserved observations, and corresponding interpretive boundaries are summarized in Table 2.

### 3.2. Source-Culture Quality Record

Among 26,071 events in the represented Annexin V/7-AAD output, 25,983 (99.66%) were viable, 86 (0.33%) were early apoptotic, no late-apoptotic events were recorded, and 2 (0.01%) were necrotic. This record provides a high-viability source-quality anchor for the workflow. Because it is one represented archived output, it is not used to estimate between-culture viability or population-level variability. Detailed Population 1/Population 2 and CD34/CD38 region values are retained in Supplementary Table S3 solely as archival analytical regions.

### 3.3. Recovered-Preparation Particle and Protein Records

The recovered preparation was represented by a heterogeneous particle-associated record with a predominant archived region at approximately 170–240 nm and by an independent immunoblot record reporting CD9, CD63, CD81, and TSG101 detection. These two qualitative layers converge on the presence of particle-associated material containing an EV-associated protein component. They do not establish a purified exosome product, quantitative particle concentration, or a specific vesicle biogenesis pathway.

### 3.4. Final Conditioned Extracellular-Environment Record

The independent aggregate cytokine summary, labeled as representing ten conditioned cultures, reported mean values of 25,320.16 pg/mL IL-10, 659.35 pg/mL IL-4, 99.61 pg/mL IL-6, and 3.01 pg/mL TNF-α. Numerically, IL-10 was the largest value in this archived final-environment record. However, because IL-10 and IL-4 were supplied during conditioning, their concentrations cannot be interpreted as evidence of de novo secretion by the source cultures. Culture-level values were not retained, so no standard deviation or inferential comparison can be calculated retrospectively. Detailed extracellular-preparation and cytokine records are provided in Supplementary Table S4. The archived evidence domains and their distinct analytical units are summarized in Figure 2.

### 3.5. Integrated Analytical Contribution and Evidence Boundary

The principal result of the integration is not a new functional phenotype but an auditable analytical architecture that preserves heterogeneous evidence without upgrading its evidentiary level. Source quality, recovered-output particle/protein records, and the final conditioned extracellular environment can be linked within one provenance chain while remaining analytically distinct. This structure prevents three common interpretive errors in retrospective extracellular-biology datasets: treating different analytical units as replicates, assigning vesicle biogenesis from incomplete compositional evidence, and converting conditioning inputs or summary-level records into endogenous functional claims.

A second result of this architecture is the explicit separation of process context from biological output. The cytokine record can be placed temporally after the conditioning sequence while IL-10 and IL-4 remain identified as exogenous process inputs; the particle and immunoblot records can be associated with the recovered preparation without converting that association into a biogenesis claim; and the single represented flow output can serve as a source-quality record without being generalized to population-level reproducibility. The integrated interpretation is therefore stronger because its claims remain narrower than the aggregate visual impression of the combined records.

This distinction also clarifies the role of negative information in the archive. The absence of raw NTA/FCS files, uncropped immunoblots, donor-level linkage, and culture-level cytokine measurements is not treated as evidence of absence of the underlying biological phenomena. Instead, it defines the level at which the present study must stop. In this sense, the evidence boundary is itself an analytical output: it identifies which questions can be answered by the preserved record and which require prospectively designed experiments.

## 4. Discussion

### 4.1. Methodological Contribution of the Retrospective Integration

The revised framing of this study is intentionally methodological. The archive does not support a claim of functional tolerogenicity, mechanistic membrane remodeling, or purified exosome identity. It does support a provenance-controlled reconstruction of how an immunoregulatory-conditioned human PBMC source, a complete recovered extracellular output, and final-environment analytical records can be connected without conflating their experimental units. In this context, the scientific contribution lies in evidence governance: this study makes explicit what each archived record supports and, equally importantly, what it does not support.

This approach is relevant to multicomponent extracellular preparations because heterogeneous archives can easily encourage overinterpretation. Particle size, EV-associated proteins, and cytokine summaries are often treated as if they were interchangeable measures of one biological entity. The present framework instead maintains the complete recovered preparation as the analytical object while using the particle and protein records only to define its documented compositional layers [8,9,10,13,14].

The practical value of this restraint is that it protects several distinctions that are routinely blurred in multicomponent systems. First, a source-cell condition is not equivalent to a product mechanism. Second, a compositional signal is not equivalent to potency. Third, an EV-associated marker pattern is not equivalent to purified-exosome identity. Finally, an aggregate final-environment measurement is not equivalent to a replicate-resolved secretion phenotype. By encoding these distinctions directly into the analytical architecture, this study converts archival limitations into explicit rules for interpretation rather than leaving them as implicit caveats.

A future laboratory cannot reproduce undocumented centrifugation forces or reconstruct missing donor linkage from the present archive, but it can reproduce the analytical rules used to classify, link, and bound heterogeneous source-to-output records. This distinction is important for development-stage evidence: reproducibility of the integration framework should not be confused with experimental replication of the historical workflow [16,17,18,19,21].

### 4.2. Position Relative to Human Tolerogenic-DC and DC-Extracellular-Output Studies

The most relevant human literature spans distinct evidentiary layers rather than one uniform experimental category. Human tolDC studies have established IL-10-dependent molecular programs and regulatory cellular phenotypes [2,7,11]. Elsayed et al. extended this logic to purified exosomes from regulatory human monocyte-derived dendritic cells generated with TGF-β/IL-10 and demonstrated recipient-cell functional effects [12]. These studies are biologically more mature than the present archive with respect to prospective functional validation, but they analyze defined cellular products or purified vesicle fractions. The present study addresses a different upstream problem: retrospective integration of the complete heterogeneous extracellular output while retaining source provenance and a biogenesis-neutral identity.

The comparison therefore places the present work upstream of direct potency or mechanism studies rather than in competition with them. Human tolDC studies establish that regulatory programming can be measured prospectively at the cellular level, and human regulatory-DC exosome studies demonstrate that a purified extracellular fraction can be tested functionally. The current archive addresses the intervening analytical question of how a complete, heterogeneous output should be described when its retained evidence is insufficient for purified-vesicle assignment or functional attribution. The novelty resides in formalizing that intermediate evidentiary layer and making its claim boundary explicit. A comparative positioning of the present study relative to selected human tolerogenic-DC and DC-extracellular-output studies is provided in Table 3.

### 4.3. Study Boundaries

The principal limitations arise directly from the retrospective nature of the archive. The records did not preserve a contemporaneous IL-4/GM-CSF-only comparator, an immunogenic-conditioning comparator, a cell-free cytokine-processing control, complete source-cell regulatory phenotyping, original NTA/FCS instrument files, uncropped quantitative immunoblots, culture-level cytokine values, or a responder-cell functional assay. Donor-level identifiers and a reliable donor count were also not retained. These limitations prevent conclusions regarding conditioning causality, between-culture reproducibility, donor dependence, particle concentration, exosome biogenesis, antigen specificity, membrane-microdomain remodeling, or functional tolerogenicity. The absence of these records is not addressed by statistical reconstruction or surrogate imputation.

These limitations are consequential, but they do not all represent missing endpoints from a single experimental design. Several belong to different experimental questions. Comparator cultures and cell-free cytokine controls would address conditioning causality; raw NTA files and quantitative immunoblots would address product characterization and reproducibility; responder-cell assays would address potency; and membrane-order or receptor-topology experiments would address mechanism. Treating all of these as mandatory outputs of one retrospective integration would collapse distinct development questions into one evidentiary tier.

For the same reason, the absence of culture-level cytokine values is handled conservatively. The archived aggregate means are retained because they are part of the documented final-environment record, but no standard deviation is reconstructed because the underlying culture-level observations are unavailable. This restriction prevents false precision and is consistent with the study-wide rule that missing replicate structure cannot be manufactured retrospectively. The same principle governs the treatment of particle size, immunoblot detection, and donor information.

### 4.4. Position Within a Modular Research Program

The broader research program is structured as a sequence of distinct evidentiary questions. Source provenance and archived analytical integration, fraction-resolved composition, functional potency, membrane-level mechanism, and antigen-specific immune responses require different experimental units, controls, and decision criteria. The present study is restricted to the provenance and archive-integration question. Functional tolerogenicity, fraction-specific activity, membrane-microdomain effects, and recipient-cell responses therefore fall outside the evidentiary scope of this retrospective analysis and require dedicated prospective designs. This separation prevents evidence generated for one scientific question from being used to support a different claim.

A modular strategy is particularly appropriate for complex extracellular preparations because identity, potency, mechanism, and specificity are not interchangeable regulatory or scientific questions. Fraction-resolved composition requires normalization and recovery accounting; potency requires predefined responder systems and appropriate controls; membrane-level mechanism requires direct biophysical or spatial assays [31]; and antigen specificity requires cognate/unrelated antigen comparisons and stability testing. Each module therefore has its own experimental unit, controls, and decision criteria. Maintaining those modules separately prevents evidence generated for one purpose from being overextended to support another. A prospective validation map for these dedicated modules is provided in Supplementary Table S5.

Within that sequence, the present study occupies the provenance and evidence-governance layer. Its role is to define the analytical object and the resolution of the historical evidence before downstream questions are tested. Functional or mechanistic endpoints require prospective controls and assay-specific designs that the current archive cannot provide. The resulting research architecture is cumulative rather than fragmentary: each experimental module adds a new evidentiary level without retrospectively upgrading the preceding one.

### 4.5. Transferability and Developmental Implications

Although the present dataset is specific to one archived human PBMC-derived workflow, the integration logic is transferable to other development archives containing heterogeneous extracellular records. The transferable element is not the numerical profile itself; it is the requirement that each measurement retain its original analytical unit, process position, and claim ceiling. This approach can be applied whenever historical source-cell, particle, protein, soluble-factor, or functional records were generated asynchronously and must later be interpreted as part of one development pathway [16,17,18,19,32].

The framework is also useful for deciding what a subsequent prospective study must add. Once the preserved evidence is mapped explicitly, the next experiment can be designed around the unresolved decision rather than around repetition of every historical measurement. For the present system, fraction-resolved recovery and quantitative characterization would address composition; normalized responder-cell assays would address potency; membrane-order and receptor-topology measurements would address mechanism; and antigen-specific challenge/rechallenge designs would address specificity and stability [33,34,35]. The retrospective integration therefore serves as a planning baseline, not as a substitute for those experiments.

Scientifically, this separation provides a disciplined route from archival identity to prospective validation. A source-to-output archive can be interpreted at the resolution it supports, while higher-order claims are reserved for experiments specifically designed to test them. Identity, composition, potency, mechanism, and antigen specificity are therefore treated as distinct evidentiary questions rather than as interchangeable outputs of a single dataset. This structure keeps conclusions anchored to the data generated for each question and limits transfer of inference across evidentiary levels. The resulting modular research-program framework is summarized in Figure 3.

## 5. Conclusions

The principal contribution of this study is methodological rather than functional. It demonstrates how heterogeneous archived evidence from an immunoregulatory-conditioned human PBMC workflow can be integrated without conflating experimental units, reconstructing unavailable data, assigning unsupported vesicle biogenesis, or inferring biological function from compositional observations. One represented high-viability source output, a qualitative particle-associated record, an archived EV-associated protein record, and an independent aggregate final-environment cytokine record can be linked within a coherent source-to-output provenance chain while retaining their distinct evidentiary limits. The resulting analytical baseline supports a biogenesis-neutral operational identity for the complete recovered preparation and defines, rather than substitutes for, the dedicated prospective studies required to test fraction-specific composition, functional immune regulation, membrane organization, and antigen-specific effects.

The broader implication is that retrospective extracellular-biology datasets need not be discarded because their raw-data preservation is incomplete, but neither should they be upgraded beyond their documentary resolution. A provenance-controlled integration can preserve useful source, composition, and process-context information while explicitly identifying the experiments required to move from analytical identity to reproducibility, potency, mechanism, and specificity. In this way, the present work defines a disciplined intermediate evidentiary layer between historical development records and prospectively validated biological claims.

## Figures and Tables

**Figure 1:**
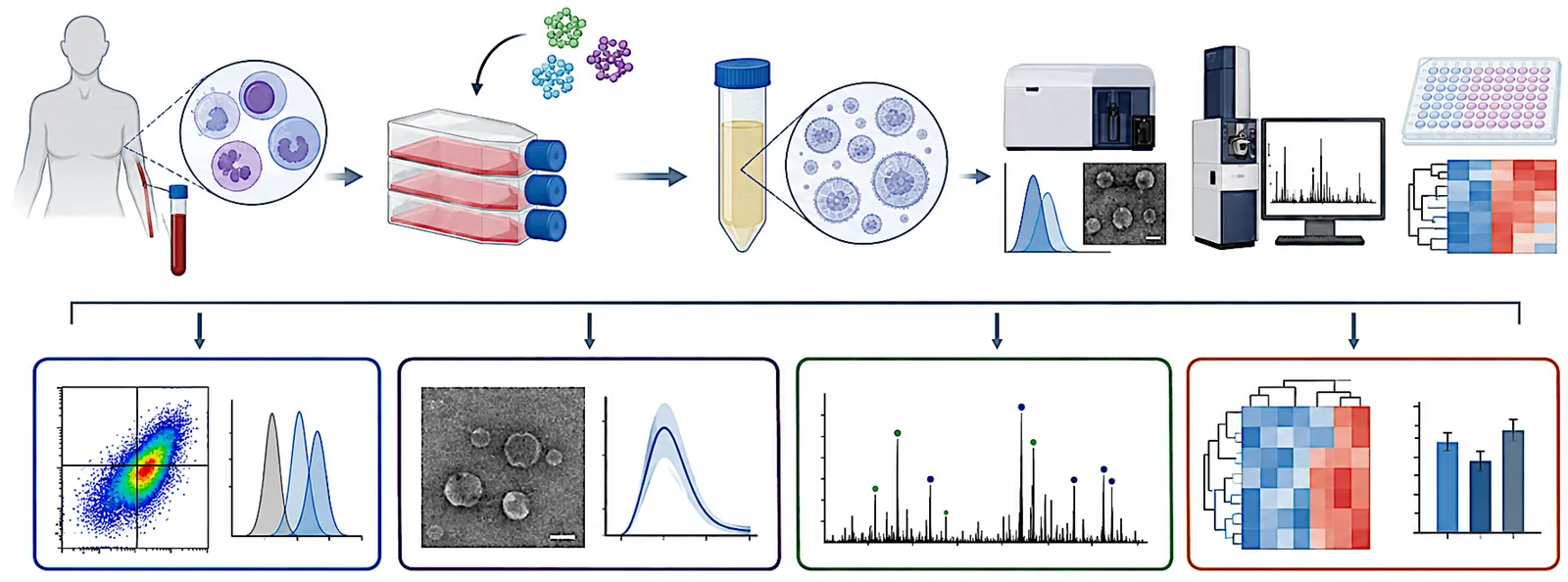
Retrospective source-to-output evidence architecture. The figure links the established human PBMC-derived source, sequential cytokine programming, extracellular recovery, and the four archived evidence domains. The lower panel defines the interpretive boundary. This is a conceptual schematic and not direct experimental imaging, quantitative particle analysis, or mechanistic evidence.

**Figure 2:**
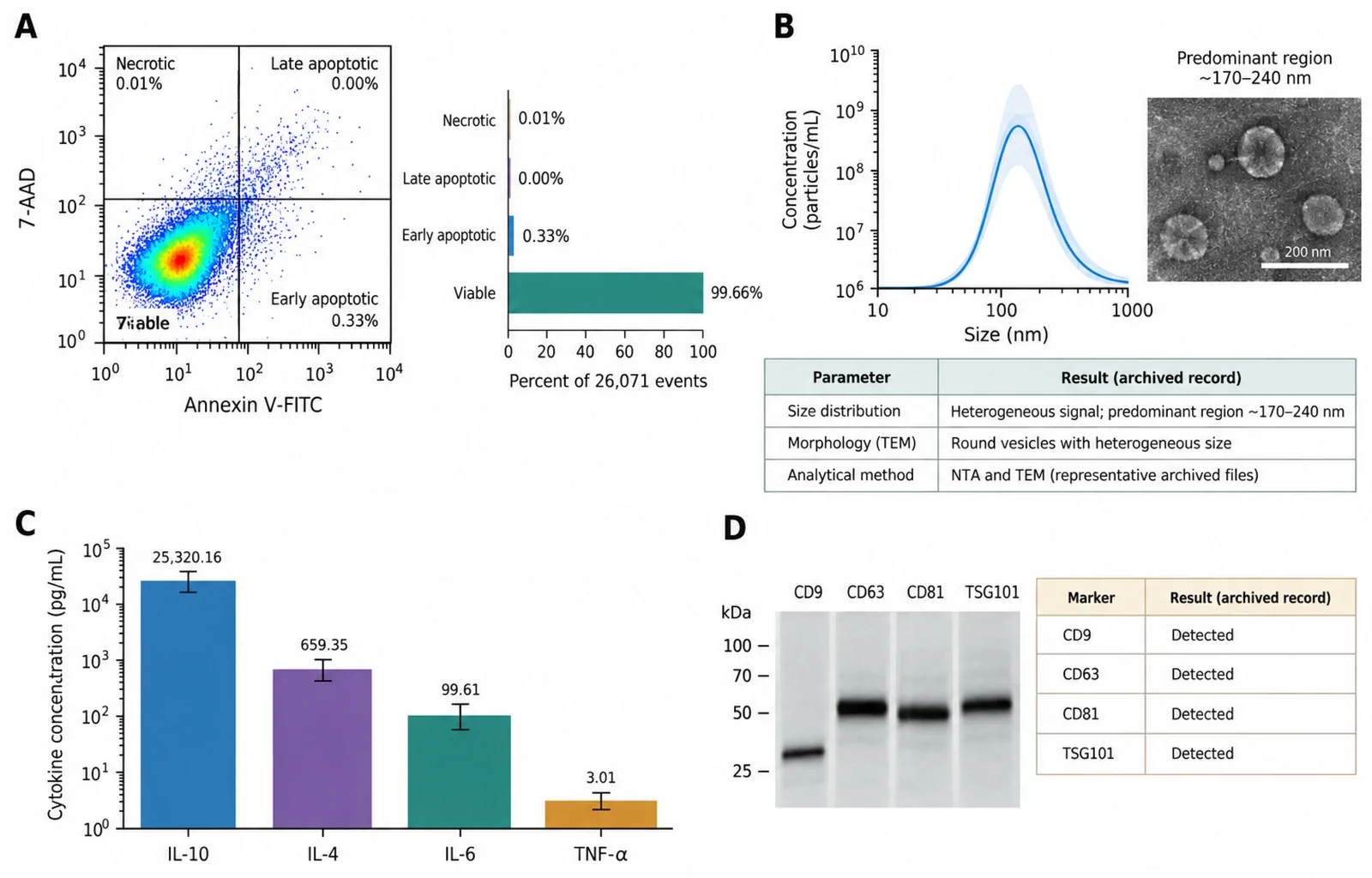
Archived evidence preserved at its original resolution. (**A**) One represented source-culture viability output. (**B**) Qualitative archived particle and protein records from the recovered preparation. (**C**) Aggregate final-environment cytokine record labeled as ten conditioned cultures; IL-10 and IL-4 were conditioning inputs. (**D**) Explicit distinction between the flow-cytometry and cytokine experimental units. The figure summarizes archived records and does not substitute for unavailable raw instrument files or replicate-level data.

**Figure 3:**
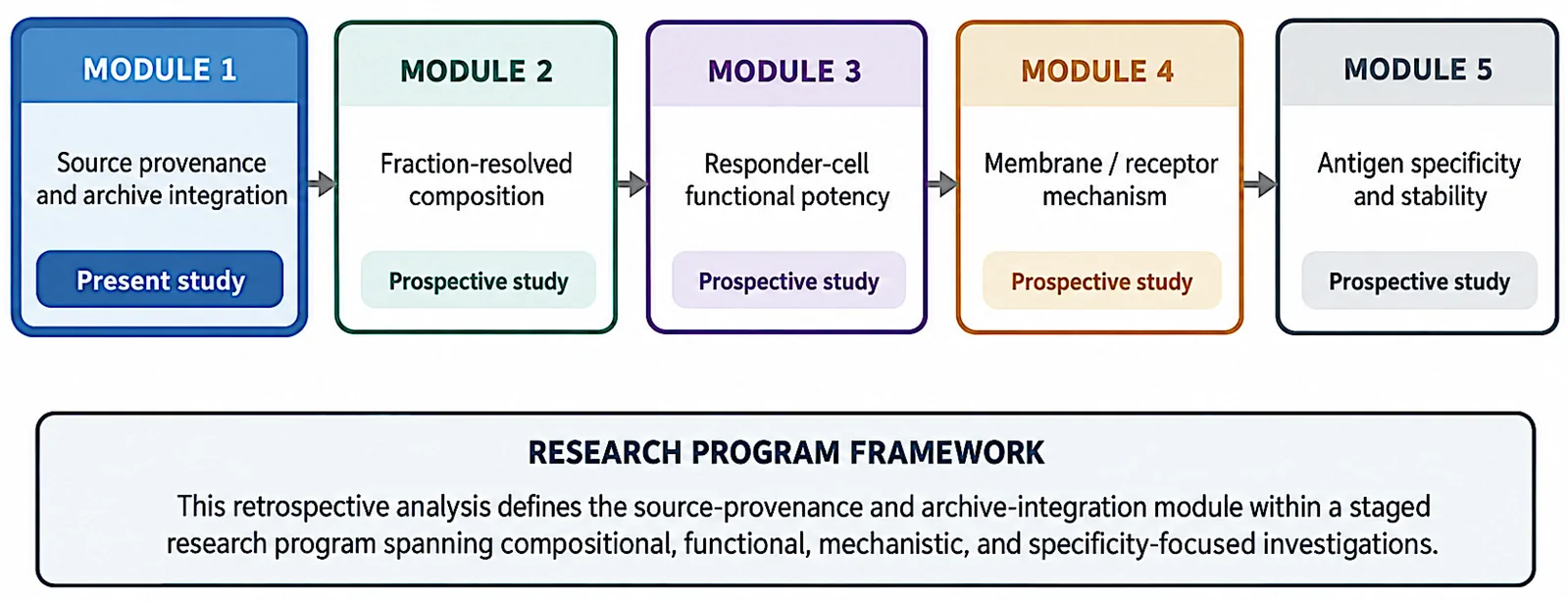
Position of the present study within a modular research program. The present manuscript is restricted to source provenance, archive integration, and evidence-boundary definition. Fraction-resolved composition, functional potency, membrane/receptor mechanisms, and antigen specificity require dedicated prospective studies. This is a conceptual research-program schematic and not experimental evidence.

**Table 1:** Stage-level workflow and archival resolution. Implementation parameters not preserved in the archive were not reconstructed.

Stage	Source Context	Documented Operation	Archived Resolution
**1**	Established human PBMC-derived cultures	Controlled in vitro maintenance	Stage and source provenance
**2**	Differentiation support	IL-4/GM-CSF-supported programming	Cytokine classes and order
**3**	Regulatory conditioning	Sequential IL-10/TGF-β1 support	Cytokine classes and order
**4**	Extracellular recovery	Serial clarification and size-based recovery	Processing classes
**5**	Retrospective integration	Flow, particle, protein, and cytokine records	Summary-level analytical units

**Table 2:** Evidence provenance and interpretive boundary. The table separates the archived analytical units and states the maximum claim supported by each record.

Domain	Archived Unit	Preserved Observation	Supports	Does not Establish
**Source viability**	One represented flow output	26,071 events; 99.66% viable	Source-quality record	Population-level reproducibility
**Particle record**	One archived qualitative NTA summary	Heterogeneous signal; predominant region ~170–240 nm	Particle-associated material	Concentration, dispersion, or vesicle biogenesis
**Protein record**	One archived band-level summary	CD9, CD63, CD81, TSG101 reported as detected	EV-associated protein component	Purity, abundance, or exosome-only identity
**Cytokine record**	Aggregate summary reported as *n* = 10 conditioned cultures	IL-10, IL-4, IL-6, TNF-α aggregate means	Final conditioned extracellular environment	De novo secretion, culture-level variance, or donor-level inference

**Table 3:** Positioning relative to selected human tolerogenic-dendritic-cell and DC-extracellular-output studies. The comparison distinguishes cellular, purified-vesicle, functional, and retrospective source-to-output evidence.

Study	Human Model/Programming	Primary Analytical Level	Functional Testing	Relevance to Present Study
**Avancini et al.** [2]	Human monocyte-derived tolDC; IL-10-dependent program	Chromatin/transcriptomic and cellular regulatory state	Yes, cellular tolerogenic activity	Human molecular precedent for IL-10-conditioned regulatory DC biology
**Dao Nyesiga et al.** [7]	Human tolDC generated with TGF-β, retinoic acid, and AhR agonism	Cell phenotype, antigen handling, immune co-culture	Yes	Human precedent for stable regulatory-cell programming
**Barran et al.** [11]	Human monocyte-derived tolDC subtypes	Cell phenotype and CD4+ T-cell response	Yes	Shows functional heterogeneity across human tolDC products
**Elsayed et al.** [12]	Human regulatory MoDCs generated with TGF-β/IL-10; purified exosomes	Purified exosome characterization and recipient-cell assays	Yes	Direct human precedent for regulatory DC-derived extracellular-vesicle function
**Present study**	Human PBMC-derived cultures; IL-4/GM-CSF then IL-10/TGF-β1	Retrospective provenance and complete-output integration	No; prospectively separated	Addresses the upstream archival-integration gap without assigning purified-vesicle identity

## Data Availability

The analytical records available for the present retrospective integration are reported within the article and its Supplementary Materials. The source archive did not preserve the original instrument-level NTA/FCS files, uncropped immunoblot images, or culture-level cytokine measurements required for retrospective replicate-level analysis. These materials are therefore not available and were not reconstructed or inferred.

## Supplementary Materials

The following supporting information can be downloaded at: https://www.mdpi.com/article/10.3390/cimb48100972/s1.

## Abbreviations

Abbreviation	Definition
CBA	Cytometric bead array
EV	Extracellular vesicle
GM-CSF	Granulocyte-macrophage colony-stimulating factor
NTA	Nanoparticle tracking analysis
PBMC	Peripheral blood mononuclear cell
TGF-β1	Transforming growth factor beta 1
7-AAD	7-aminoactinomycin D
