# Supplementary material for: Source-to-Output Integration of Archived Evidence from an Immunoregulatory-Conditioned Human PBMC Extracellular Preparation
Source: Curr Issues Mol Biol. 2026 Sep 22;48(10):972. doi: 10.3390/cimb48100972 (PMC13703046; doi:10.3390/cimb48100972)
Supplement: Supplementary file 1 [file cimb-48-00972-s001.zip › 02_Supplementary_Materials_CIMB_4521509/Table_S1.pdf]

Supplementary Table S1

Archived domain	Record retained	Analytical unit	Inclusion rule	Main-manuscript role
Source viability	Annexin V/7-AAD summary	One represented output; 26,071 events	Included because it is unambiguously linked to the source workflow and retains an interpretable unit	Source-quality anchor
Cell-associated regions	Parent and CD34/CD38 region summaries	Archived gate summaries	Included for provenance only; no phenotype inferred	Supplementary traceability
Particle record	Qualitative NTA/NanoSight summary	One archived qualitative summary	Included at preserved qualitative resolution	Particle-associated layer
Protein record	Band-level immunoblot summary	One archived qualitative summary	Included at preserved qualitative resolution	EV-associated protein layer
Cytokine record	Aggregate multiplex summary	Reported n = 10 conditioned cultures	Included as an independent aggregate final-environment record	Process-aware context layer

Table S1. Archive-selection and analytical-provenance matrix. No record was excluded on the basis of result direction or magnitude.
