# Supplementary material for: Source-to-Output Integration of Archived Evidence from an Immunoregulatory-Conditioned Human PBMC Extracellular Preparation
Source: Curr Issues Mol Biol. 2026 Sep 22;48(10):972. doi: 10.3390/cimb48100972 (PMC13703046; doi:10.3390/cimb48100972)
Supplement: Supplementary file 1 [file cimb-48-00972-s001.zip › 02_Supplementary_Materials_CIMB_4521509/Table_S3.pdf]

Supplementary Table S3

Analytical population	Number/fraction	Percentage or secondary-region output	Interpretive status
Viable	25,983 of 26,071	99.66%	Source-quality record
Early apoptotic	86 of 26,071	0.33%	Archived minor outcome
Late apoptotic	0 of 26,071	0.00%	Archived minor outcome
Necrotic	2 of 26,071	0.01%	Archived minor outcome
Population 1	30.34% of parent gate	98.74% in CD34/CD38 region	Archived analytical region; no phenotype assigned
Population 2	56.93% of parent gate	98.63% in CD34/CD38 region	Archived analytical region; no phenotype assigned

Table S3. Detailed archived source-culture numerical outputs. Population 1 and Population 2 are retained only as neutral analytical labels because original gating templates and phenotypic validation records were not preserved.
