# Supplementary material for: Source-to-Output Integration of Archived Evidence from an Immunoregulatory-Conditioned Human PBMC Extracellular Preparation
Source: Curr Issues Mol Biol. 2026 Sep 22;48(10):972. doi: 10.3390/cimb48100972 (PMC13703046; doi:10.3390/cimb48100972)
Supplement: Supplementary file 1 [file cimb-48-00972-s001.zip › 02_Supplementary_Materials_CIMB_4521509/Table_S2.pdf]

Supplementary Table S2

Stage	Source context	Documented operation	Material carried forward	Preserved resolution
1	Established human PBMC-derived cultures	Controlled in vitro maintenance	Source cultures	Source and stage provenance
2	Differentiation support	IL-4/GM-CSF-supported programming	Differentiating cultures	Cytokine classes and order
3	Regulatory conditioning	Sequential IL-10/TGF-β1 support	Conditioned cultures	Cytokine classes and order
4	Extracellular recovery	Serial clarification and size-based recovery	Complete extracellular preparation	Processing classes only
5	Retrospective integration	Analytical record linkage	Archived evidence domains	Summary-level units

Table S2. Stage-level workflow and preserved archive resolution. Exact concentrations, exposure durations, recovery parameters, and storage conditions were not retained and were not reconstructed.
