# Supplementary material for: Source-to-Output Integration of Archived Evidence from an Immunoregulatory-Conditioned Human PBMC Extracellular Preparation
Source: Curr Issues Mol Biol. 2026 Sep 22;48(10):972. doi: 10.3390/cimb48100972 (PMC13703046; doi:10.3390/cimb48100972)
Supplement: Supplementary file 1 [file cimb-48-00972-s001.zip › 02_Supplementary_Materials_CIMB_4521509/Table_S5.pdf]

Supplementary Table S5

The following modules are deliberately separated from the present retrospective study. They represent dedicated prospective research questions rather than missing outcomes of the archived analysis.

Prospective question	Minimum comparison	Primary readouts	Decision enabled
Is activity dependent on the complete preparation?	Complete vs EV-enriched vs EV-depleted vs soluble fractions	Normalized responder-cell and cytokine outcomes	Multicomponent vs fraction-specific activity
Are retained cytokines process-related or associated with recovered fractions?	Cell-free medium, washout, and particle-depleted controls	IL-10, TGF-β1, and IL-4 mass balance	Process-component assignment
Does the preparation alter membrane organization?	Vehicle vs conditioned preparation	Membrane order, GM1 mapping, receptor nanoclustering	Direct microdomain evidence
Is the effect functionally regulatory?	Activated responders ± preparation with viability control	Proliferation, CD69/CD25, Treg panel, cytokines	Functional regulatory effect
Is any regulatory effect antigen-specific and stable?	Cognate vs unrelated antigen; inflammatory rechallenge	Hyporesponsiveness, preserved unrelated response, stability	Antigen-specific classification

Table S5. Prospective validation map. None of these modules is presented as completed evidence in the revised manuscript.
