# Supplementary material for: Source-to-Output Integration of Archived Evidence from an Immunoregulatory-Conditioned Human PBMC Extracellular Preparation
Source: Curr Issues Mol Biol. 2026 Sep 22;48(10):972. doi: 10.3390/cimb48100972 (PMC13703046; doi:10.3390/cimb48100972)
Supplement: Supplementary file 1 [file cimb-48-00972-s001.zip › 02_Supplementary_Materials_CIMB_4521509/Table_S4.pdf]

Supplementary Table S4

Domain	Directly documented observation	Interpretation used in revised manuscript	Explicit boundary
NTA/NanoSight	Heterogeneous signal; predominant region ~170-240 nm	Particle-associated material is present	No concentration, quantitative dispersion, or biogenesis assignment
Immunoblot	CD9, CD63, CD81, and TSG101 reported as detected	EV-associated protein component	No abundance, purity, or exosome-only identity
Cytokines	Aggregate means: IL-10 25,320.16; IL-4 659.35; IL-6 99.61; TNF-α 3.01 pg/mL	Final conditioned extracellular-environment record	IL-10/IL-4 are conditioning inputs; no de novo secretion or SD inferred

Table S4. Detailed extracellular-preparation and cytokine records. The cytokine summary was labelled as representing ten conditioned cultures; culture-level values were not retained.
